# The green bad omen in blood smear and the potential of blood purification therapy: A case report

**DOI:** 10.1097/MD.0000000000036205

**Published:** 2024-01-05

**Authors:** Yanhui Chen, Wenpeng Ni, Guanghong Gu

**Affiliations:** a Clinical Laboratory, Boai Hospital of Zhongshan Affiliated to Southern Medical University, Zhongshan, P.R. China; b Clinical Laboratory, Zhongshan Torch Development Zone People’s Hospital, Zhongshan, P.R. China.

**Keywords:** blood purification therapy, disseminated intravascular coagulation, green inclusion, liver damage, peripheral blood smear

## Abstract

**Rationale:**

Green inclusions (GI) are distinct morphological features found in phagocytic cells like neutrophils and monocytes. These intracellular structures exhibit bright green color with unclear boundaries, and their origin and clinical significance are still not fully understood. GI carriers, often middle-aged to elderly with liver dysfunction, face higher mortality rates, earning them the nickname “inclusions of death.” This report presents a rare GI-related pediatric case, demonstrating a favorable response to blood purification therapy.

**Patient concerns:**

A 10-year-old girl was admitted with recurrent fever, abdominal pain, and neurological symptoms, culminating in a transient cardiac arrest. Blood tests revealed multi-organ injury and a high risk of disseminated intravascular coagulation, while peripheral blood smear detected GI within neutrophil cytoplasm.

**Diagnosis:**

The patient was diagnosed with acute necrotizing encephalopathy, severe sepsis, and multiple organ failure.

**Interventions and outcomes:**

After receiving multiple sessions of blood purification therapy, peripheral blood GI levels markedly decreased, accompanied by improvements in various laboratory parameters and signs of neurological recovery. Unfortunately, due to financial constraints, the family opted to transfer the patient back to their local hospital, where she succumbed shortly after discharge.

**Lessons:**

This case underscores the complexities in managing GI-related pediatric cases. Moreover, it emphasizes the potential benefits of blood purification therapy in such scenarios. Notably, this study highlights a potential correlation between the level of GI in peripheral blood and disease severity, particularly in pediatric cases. While these findings hold clinical significance for the treatment and management of GI-related patients, further research focusing on middle-aged and elderly individuals is imperative to elucidate the fundamental relationship between peripheral blood GI quantity and clinical presentation and to evaluate the efficacy of blood purification in GI-related cases.

## 1. Introduction

Green inclusions (GI) is typically observed as a morphological feature found exclusively in cells with phagocytic capacity, such as neutrophils and monocytes, while their presence in lymphocytes has not been reported. These intracellular inclusions, easily recognizable morphologically, appear as bright green structures within the cytoplasm and exhibit either blurry boundaries or round/irregular shapes.^[1]^

The formation mechanism, composition, and clinical significance of GI remain unclear. Hodgson et al, based on Periodic Acid-Schiff staining and Zeihl-Neelsen staining results, proposed that GI may be lysosomal degradation products formed after neutrophils or monocytes phagocytize tissue injury products. They further speculated that GI formation could be attributed to the phagocytic digestion of lipofuscin released by acute liver damage-induced hepatocyte necrosis by monocyte macrophages and Kupffer cells.^[2]^ Previous reports have shown that carriers of GI are often middle-aged to elderly individuals with concurrent liver dysfunction, exhibiting a significantly high clinical mortality rate. Consequently, some scholars have referred to GI as “inclusions of death.”^[1,3]^ In this report, we present a rare case of a pediatric patient who demonstrated an effective response to treatment, but unfortunately, the outcome was disappointing.

## 2. Case report

A 10-year-old girl presented with recurrent fever, reaching a maximum temperature of 39.0°C, accompanied by chills and abdominal pain. She sought medical treatment at a local hospital, where a complete blood cell count revealed “white blood cells 5.97 (4.30–11.30) × 10^9^/L, neutrophil percentage 66.50% (31.0–70.0%); red blood cells 6.06 (4.20–5.70) × 10^12^/L, hemoglobin 124.0 (118.0–156.0) g/L, platelets 241.0 (167.0–453.0) × 10^9^/L.” Despite treatment (including oral antipyretic medication, details unspecified), the patient continued to experience recurrent fever and exhibited symptoms such as lethargy. Subsequently, she developed unresponsiveness, fixed gaze, generalized limb stiffness, urinary and fecal incontinence, and vomiting, leading to her transfer to our hospital for further management.

Given the severity of patient’s condition, she was transported to our hospital by an ambulance. Upon the arrival of the medical team, the patient was unresponsive, had irregular and labored breathing, and moaned, with a significant amount of frothy oral secretions. Physical examination revealed reduced level of consciousness with delayed pupillary light reflexes. She was immediately placed under cardiac monitoring, intubated, connected to a ventilator, and administered fluids before being transported. During transit, the patient had a heart rate of 130.0 beats per minute and blood oxygen saturation levels ranging from 95.0% to 100.0%. Upon arrival at the hospital, the cardiac monitor indicated a drop in heart rate to 60.0 beats per minute, no audible heart sounds and unpalpable arterial pulses, signaling cardiac arrest. Following resuscitation measures, her body temperature was 35.10°C, and her Glasgow Coma Scale score was E1VtM1.

She was maintained on dopamine at 12.50 μg/kg/min, epinephrine at 0.30 μg/kg/min, and norepinephrine at 0.30 μg/kg/min, with a heart rate of 111.0 beats/min and blood pressure of 60.0/40.0 mm Hg. Both pupils had a diameter of 4.0 mm with absent light reflex. Heart sounds were muffled but regular, and the extremities were cool with a capillary refill time of 4.0 seconds.

**Figure 1.**
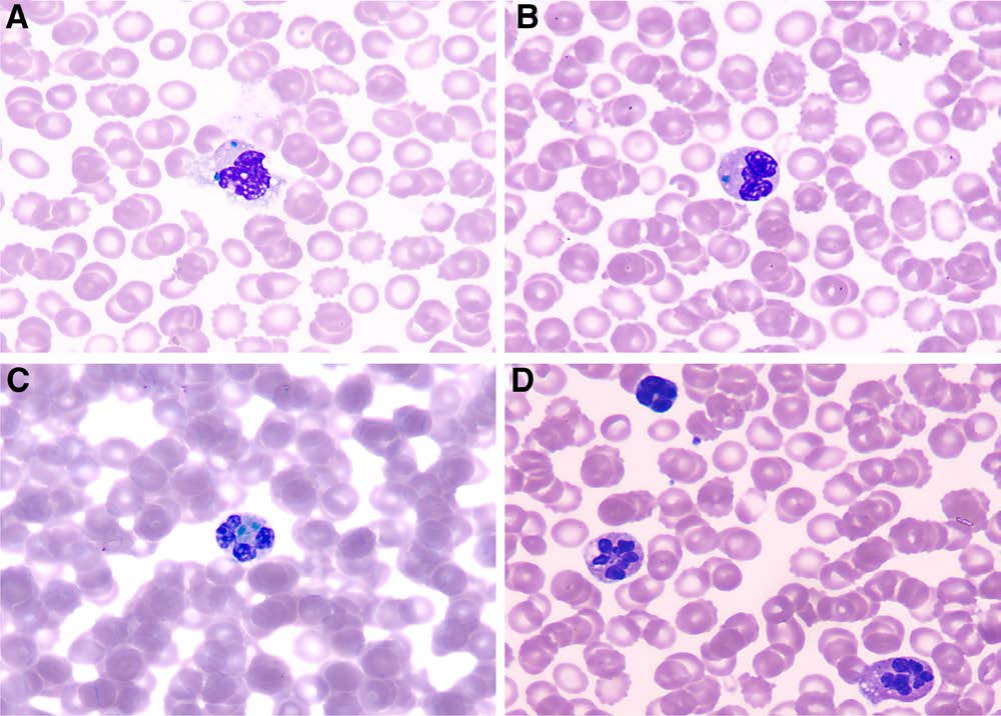
Images from the Peripheral blood smear: GI in neutrophils (A–C), multi-lobed neutrophils and degenerated cell (D). (Wright and Giemsa staining, ×1000). GI = Green inclusions.

Laboratory tests revealed severe coagulation dysfunction in the patient, with the following results: prothrombin time 109.30 seconds (9.0–13.20), activated partial thromboplastin time 133.60 seconds (22.50–34.50), Fibrinogen < 0.25 g/L (1.80–4.0), thrombin time > 160.0 seconds (14.0–21.0), D-Dimer 430.79 μg/mL (0–0.50). Considering the clinical manifestations (evidence of bright red gastric fluid on gastric tube aspiration), disseminated intravascular coagulation was suspected. The patient was treated with low molecular weight heparin (1.0 mg/kg Bid) for anticoagulation and received 600.0 mL of fresh frozen plasma. Complete blood cell count showed: leukocyte 10.08 × 10^9^/L, erythrocyte 7.03 × 10^12^/L, hemoglobin 127.0 g/L, platelet 24.0 × 10^9^/L, neutrophil percentage 57.70%, red blood cell volume distribution width-CV 16.70 (11.0–16.0), mean corpuscular volume 59.40 (77.0–92.0) fl. Peripheral blood smear showed the presence of GI in 10.0% of neutrophil cytoplasm (Fig. 1A–C), as well as multi-lobed neutrophils, apoptotic and degenerated cells (Fig. 1D), indicating a poor prognosis. The blood gas analysis results indicated a pH of 7.15 (7.35–7.45), a partial pressure of carbon dioxide of 26.0 (35.0–45.0) mm Hg, lactate concentration of 13.20 (0.50–1.70) mmol/L, a base excess of −19.0 (−3.0 to 3.0) mmol/L, and a blood glucose level of 2.60 (3.90–6.10) mmol/L. These findings raised concerns of severe metabolic acidosis and volume depletion. Consequently, sodium bicarbonate was administered to correct the acidosis, intravenous fluid resuscitation was initiated for volume expansion, and 50.0 mL of 10.0% glucose was administered intravenously. Other laboratory findings included urea 9.39 (2.50–6.50) mmol/L, creatinine 649.0 (27.0–66.0) μmol/L, alanine aminotransferase 95.0 (8.0−30.0) U/L, aspartate aminotransferase 454.0 (18.0−45.0) U/L, creatine kinase (CK) 316.0 (40.0−200.0) U/L, CK-MB 149.30 (0−33.0) U/L, lactate dehydrogenase 2465.0 (120.0−250.0) U/L, total protein 41.90 (65.0−84.0) g/L, albumin 27.30 (39.0−54.0) g/L, adenosine deaminase 123.0 (0−25.0) U/L, positive for influenza antigen, interleukin > 5000.0 (0−7.0) pg/mg, N-terminal pro-B-type natriuretic peptide 3547.0 (0−300.0) pg/mL, and procalcitonin 56.92 (0−0.05) ng/mL. Ultimately, we consider the patient to have acute necrotizing encephalopathy, severe sepsis, and multiple organ dysfunction. Clinical management included the administration of methylprednisolone (15.0 mg/kg) for anti-inflammatory purposes and immunomodulation with immunoglobulin (0.70 g/kg). We planned to initiate dehydration and intracranial pressure reduction therapy once hemodynamics were relatively stable.

**Figure 2.**
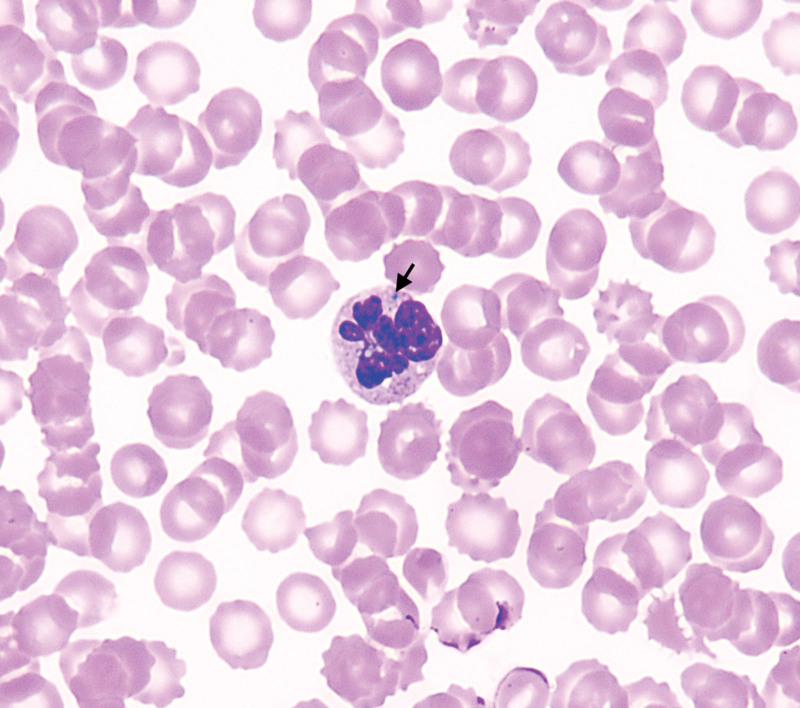
Images from the Peripheral blood smear: after treatment, GI in the peripheral blood neutrophils of patients not only decreased in number, but also became difficult to detect. (Wright and Giemsa staining, ×1000). GI = Green inclusions.

After a series of clinical treatments, including fluid resuscitation, anti-inflammatory therapy, anti-infective therapy, and blood product transfusions, the patient’s hemodynamic status remained unsatisfactory, and there was no significant improvement in multiple organ dysfunction. Therefore, after a multidisciplinary consultation, we decided to initiate blood purification therapy (combined use of hemodialysis and hemoperfusion) for the patient. The blood flow rate was set at 50.0 to 150.0 mL/min, and 400.0 mL of fresh frozen plasma was infused during each treatment session, which lasted for 2.50 hours and was performed every other day. After 2 sessions of treatment, the patient regained consciousness, and there was a significant improvement in her condition. The platelet count increased to 72.0 × 10^9^/L, and the levels of alanine aminotransferase (35.0 U/L), aspartate aminotransferase (88.0 U/L), creatinine (83.0 μmol/L), urea (5.42 mmol/L), lactate dehydrogenase (842.0 U/L), CK (102.0 U/L), CK-MB (76.0 U/L), procalcitonin (25.09 ng/mL), interleukin (753.0 pg/mg), D-Dimer (55.43 μg/mL), and the percentage of GI-positive cells (1.0%) in peripheral blood and the number and size of GI in the cytoplasm (Fig. 2) all significantly decreased. However, when we were preparing for the next phase of treatment, the patient’s family strongly requested to return to their local hospital for further treatment due to financial reasons. Subsequently, through follow-up, we learned with great regret that the patient passed away on the fifth day after discharge.

## 3. Discussion

GI are enigmatic intracellular structures observed predominantly in phagocytic cells like neutrophils and monocytes. Despite their distinct morphological characteristics, the origin, composition, and clinical implications of GI remain largely unknown.^[1]^ These green inclusions have garnered attention primarily due to their association with severe liver dysfunction and high mortality rates among middle-aged and elderly individuals, earning them the ominous moniker “inclusions of death.” This report presents a unique case of GI in a pediatric patient who exhibited a favorable response to blood purification therapy, shedding light on the complexities surrounding GI-related conditions.

The emergence of GI in peripheral blood smears often raises concerns about the patient’s prognosis, and previous studies have hinted at a strong correlation between GI presence and acute liver failure, severe infections, and poor outcomes. Notably, GI-positive cells have been identified in cases of acute liver failure caused by various pathogens, including Streptococcus pneumoniae, Clostridium perfringens, Escherichia coli, Enterococcus, Plasmodium falciparum, Aspergillus fumigatus, and even in the context of acute liver failure associated with SARS-CoV-2.^[4–7]^ Patients with acute liver injury who present GI-positive cells typically experience a rapid escalation in transaminase levels, worsening clinical conditions within a short timeframe, and alarmingly high mortality rates.^[8]^

However, there are exceptions to this dire prognosis, with reports suggesting that GI-positive cells can disappear, transaminase levels can decrease, and patients can recover, particularly when the underlying condition is effectively managed, as seen in middle-aged individuals with liver failure due to infections after antibiotic treatment.^[9]^ The variability in patient outcomes highlights the critical role of underlying health conditions and effective clinical interventions. Cases like that of a patient with renal cancer and early metastasis who succumbed within hours of the discovery of GI-positive cells and a contrasting scenario of a high-altitude fall patient with multiple injuries who survived emphasize this variability.^[10,11]^

The case we present here involves a pediatric patient with GI-positive cells and a relatively better initial physical condition compared to most elderly GI-positive cases. However, suboptimal initial management prior to her transfer to our hospital significantly complicated her condition. Despite our team’s concerted efforts to stabilize her health, the unfortunate outcome was influenced by family-related decisions.

In summary, this case highlights the dynamic nature of GI-positive cells in peripheral blood smears, with their presence fluctuating alongside the progression or improvement of the underlying disease. The percentage of GI-positive cells could potentially serve as an indicator of disease severity and contribute to prognostic assessments. Therefore, we emphasize the importance of timely communication between laboratory physicians and clinical teams upon the detection of GI-positive cells and advocate for continuous monitoring of peripheral blood smears. Furthermore, our experience suggests that blood purification therapy may offer temporary relief in GI-related cases, although further validation through a larger sample of clinical cases is warranted to establish its efficacy.

In conclusion, this case adds to the growing body of knowledge about GI and underscores the need for ongoing research to unravel the mysteries surrounding these enigmatic inclusions, especially in the context of middle-aged and elderly individuals with liver dysfunction. Such investigations hold the promise of improving the diagnosis, management, and outcomes of GI-related condition, ultimately reducing the burden of these patients and their families.

## Author contributions

**Data curation:** Yanhui Chen, Wenpeng Ni.

**Formal analysis:** Wenpeng Ni.

**Investigation:** Yanhui Chen.

**Writing – original draft:** Wenpeng Ni.

**Writing – review & editing:** Guanghong Gu.
